# Behaviour and burnout in medical students

**DOI:** 10.3402/meo.v19.25209

**Published:** 2014-08-25

**Authors:** Jo Cecil, Calum McHale, Jo Hart, Anita Laidlaw

**Affiliations:** 1 School of Medicine, University of St Andrews, St Andrews, UK; 2 Manchester Medical School, University of Manchester, Manchester, UK

**Keywords:** burnout, medical students, diet, physical activity, alcohol, lifestyle

## Abstract

**Background:**

Burnout is prevalent in doctors and can impact on job dissatisfaction and patient care. In medical students, burnout is associated with poorer self-rated health; however, it is unclear what factors influence its development. This study investigated whether health behaviours predict burnout in medical students.

**Methods:**

Medical students (*n*=356) at the Universities of St Andrews and Manchester completed an online questionnaire assessing: emotional exhaustion (EE), depersonalisation (DP), personal accomplishment (PA), alcohol use, physical activity, diet, and smoking.

**Results:**

Approximately 55% (54.8%) of students reported high levels of EE, 34% reported high levels of DP, and 46.6% reported low levels of PA. Linear regression analysis revealed that year of study, physical activity, and smoking status significantly predicted EE whilst gender, year of study, and institution significantly predicted DP. PA was significantly predicted by alcohol binge score, year of study, gender, and physical activity.

**Conclusions:**

Burnout is present in undergraduate medical students in the United Kingdom, and health behaviours, particularly physical activity, predict components of burnout. Gender, year of study, and institution also appear to influence the prevalence of burnout. Encouraging medical students to make healthier lifestyle choices early in their medical training may reduce the likelihood of the development of burnout.

Burnout is a measure of physical and psychological exhaustion and mental distress catalysed primarily by occupational and professional demands. It is characterised by heightened levels of emotional exhaustion (EE) and depersonalisation (DP) (described as emotional indifference and the dehumanisation of the client or patient), and a decreased perception of personal accomplishment (PA) (1). Burnout is commonly found in individuals working within human services and recent studies estimate the prevalence of burnout in American doctors to be around 40% (2, 3), although some have found it to be as high as 76% in internal medicine residents (4). The cause of burnout is complex and unclear; however, it is becoming increasingly evident that many students begin to experience burnout in medical school, with prevalence rates of around 49% in medical students in the USA and 28–61% in Australia (5).

Burnout and stress are symptomatically similar, with burnout attributed specifically to occupational stressors (6). McManus and colleagues (7) proposed that there is a cyclical relationship between stress and EE, suggesting that heightened levels of stress and poor coping strategies may be key contributors in the development of burnout. Medical school is challenging and previous work suggests that a number of factors, from academic pressures and educational debt, to personal life events, gender, learning environment and exposure to human suffering, contribute to heightened levels of stress and poor mental health in medical students, including burnout 8–11) . Additional research has also shown that maladaptive lifestyle and health behaviours as a means of coping with stress are prevalent among young people and university students, with studies suggesting associations between stress, alcohol consumption (12, 13), unhealthy diets (14, 15), and reduced physical activity (16).

Generally, it has been shown that health beliefs and behaviours are sub-optimal in European university students (17). In addition, alcohol consumption has been found to be higher in medical students than in age-matched samples in the general population (18, 19) and increased alcohol intake has also been associated with higher levels of distress, anxiety, and exam and work pressures in medical students (20, 21). Other studies have found physical inactivity, unhealthy diets, and smoking to be an issue in university and medical student samples 22–25).

Research has identified associations between burnout and lifestyle health behaviours. Gorter and colleagues (26) noted that Dutch dentists who reported high levels of burnout consumed more alcohol and had less healthy diets than those reporting lower levels of burnout. More recently, Gerber and colleagues (27) proposed that interventions to increase physical activity can help to improve the symptoms of burnout.

Poor lifestyle, poor health behaviours, and high levels of burnout have been reported in medical students; however, few studies have directly investigated the relationship between burnout, lifestyle, and health behaviours in a medical student population, with most work focusing on trainee doctors (28, 29). This study investigates the prevalence of burnout and potential association of burnout with habitual diet, physical activity, alcohol consumption, and smoking in medical students from two medical schools (St Andrews & Manchester) in the United Kingdom. More specifically this study asks:

1. Is burnout prevalent in a sample of medical students from two UK medical schools?
2. Does the prevalence of burnout vary with age, gender, year of study, and institution attended?
3. Do medical students’ lifestyle and health behaviours vary by gender, year of study, and institution?
4. Do medical students’ lifestyle and health behaviours (alcohol consumption, physical activity, diet, and smoking) predict burnout component scores?

## Methods

### Participants

All undergraduate medical students at both St Andrews and Manchester Universities were invited to participate in the study. St Andrews Medical School offers a 3-year BSc medicine degree, after which students typically move to a guaranteed place at another medical school (including the Manchester Medical School or one of the other medical schools in Scotland) to complete their medical studies. Manchester Medical School offers a 5-year MBChB degree course.

### Data collection

Consenting participants were surveyed electronically from March to May 2012. The survey consisted of four separate and validated tools used to assess burnout (30) alcohol consumption (31), physical activity levels (32), and eating habits (33). Demographic data were also collected, including age, gender, year of study, and institution. Smoking status was also recorded.

#### Maslach Burnout Inventory

The Maslach Burnout Inventory (MBI) is a standard measure of burnout (30), commonly used in medical student populations (10, 35, 36). It contains 22 questions asking the participant to rate how often they experience various feelings. Each question has a seven-point response scale (1: never, 2: a few times a year or less, 3: once a month or less, 4: a few times a month, 5: once a week, 6: a few times a week, 7: everyday), and is designed to measure three components of burnout; EE, DP, and PA. EE are characterised by feelings of emotional overextension as a result of one's work. DP is characterised by emotional indifference and the dehumanisation of the recipients of one's services. Low PA is characterised by feelings of occupational stagnation, incompetence and underachievement (1). Scores in each of the three subscales were categorised into high, average or low scores according to ‘medicine’ cut-offs detailed in the MBI manual (6). High EE was defined as scoring ≥27, high DP≥10, and low PA≤33. Participants were identified as having burnout if they scored high in EE (≥27) and DP (≥10).

#### Alcohol Use Questionnaire

The Alcohol Use Questionnaire (AUQ) measures alcohol consumption (31, 36) and has been used previously in university student and young adult populations 37–39). The 12-item questionnaire assesses the frequency and quantity of alcohol consumption within the last six months. Alcohol intake and binge scores were calculated from this data (31).

#### International Physical Activity Questionnaire

The International Physical Activity Questionnaire (IPAQ) has been recognised as a reliable tool for providing estimates of an individual's general level of physical activity (32). It has been validated internationally and in university student populations (40, 41). This study used the short-form IPAQ which asked participants to estimate how often they had taken part in vigorous and moderate exercise as well as how much time they had spent walking and sitting over the last 7 days. The questionnaire provides information with regards to exercise frequency (times per week and duration of time spent) and intensity (low, moderate, high). Metabolic equivalents (METs/week) were also calculated from responses, giving a single quantifiable score that represented each participant's level of physical activity and each participant was categorised as having a high, moderate, or low level of physical activity using the IPAQ scoring protocol (42).

#### Food Frequency Questionnaire

The Food Frequency Questionnaire (FFQ) was used to assess how often participants typically ate listed foods, allowing their habitual dietary behaviour to be examined. It was developed as a means of replacing the time-consuming diet diary method and has proven itself as a reliable alternative (33) and variations have been used in populations of young people and health professionals (43, 44). Using a short-form version of the FFQ (33), participants estimated how often they consumed each of 36 different food options on a six-point scale, ranging from ‘two or more times daily’ to ‘rarely/never’. Seven food groups of interest were constructed from these options: Fruit, Vegetables, Fruit & Vegetables, High Calorie Drinks, Sweet Foods (foods high in fat and sugar, e.g., chocolate, cakes, sweets, and candies), Savoury Foods (foods high in fat, e.g., fried foods, cheese-based foods, butter, margarines, and oils), and Savoury Snacks (crisps and similar foods, high in fat and salt). Total scores were calculated for each food group to identify frequency of consumption.

### Ethics

This study was reviewed and approved by the University of St Andrews and University of Manchester research ethics committees.

### Analysis

Descriptive summary statistics were conducted to estimate the prevalence of burnout in the student sample and to assess alcohol intake, levels of physical activity, and food frequency. Analysis of variance (ANOVA) was used to examine differences in burnout, alcohol intake, physical activity, food frequency and smoking between student genders, and year of study. Backward multiple linear regression analysis was conducted for each of the three MBI component scores to examine the relationship between burnout and lifestyle behaviours. All predictors were entered into the model initially and successively removed if *p*≥0.10 until a final model was produced. All analysis was conducted using SPSS 19. Statistical significance was set at *p*≤0.05.

## Results

### Participation and sample

A total of 2,647 students were invited to take part via an advert on their virtual learning environments, 471 medical students at the University of St Andrews and 2,176 medical students at the University of Manchester. Four hundred and seven participants were recruited and submitted an online questionnaire. Fifty-one questionnaires were removed from the analysis because of incomplete MBI questionnaires leaving 356 questionnaires. The usable data response rate from St Andrews students was 27.2% (*n*=128), whilst for Manchester students it was 10.5% (*n*=228) of the possible sample.

Participant demographic information is shown in Table 1. More female medical students (65.8%, *n*=232) than males (34.8%, *n*=124) participated. The majority of participants (90.2%, *n*=321) were between the ages of 18 and 23, and 79.7% (*n*=284) of participants were in the first, second, or third year of their medical studies.

**Table 1:** Participant demographics

Variable	*n*	% of respondents
Institution		
St Andrews only	128	36
Manchester only	195	54.8
St Andrews then Manchester	27	7.6
Other University then Manchester	6	1.7
Gender		
Male	124	34.8
Female	232	65.2
Age group		
18–23	321	90.2
24–29	30	8.4
≥30	5	1.4
Year of study		
First	141	39.6
Second	77	21.6
Third	66	18.5
Fourth	47	13.2
Fifth	25	7

### Burnout prevalence

More than half of the participants (54.8%) reported experiencing high levels of EE, 34% reported high levels of DP and 46.6% reported low levels of PA (Table 2). Overall, 26.7% of participants met the criteria to be considered ‘burned out’.

**Table 2:** MBI scoring for components of burnout and burned out prevalence by institution(s) attended, gender and year of study

	Emotional exhaustion (EE)		Depersonalisation (DP)		Personal accomplishment (PA)		
				
	Mean (SD)	*n* (%) high EE score	Mean (SD)	*n* (%) high DP score	Mean (SD)	*n* (%) low PA score	Burned out *n* (%)
All participants (*n*=356)	28.26 (10.08)	195 (54.8)	9.08 (4.30)	121 (34)	38.07 (9.39)	166 (46.6)	95 (26.7)
Institution							
St Andrews (*n*=128)	28.59 (10.39)	70 (54.7)	9.26 (4.47)	50 (39.1)	38.16 (9.72)	60 (46.9)	39 (30.5)
Manchester (*n*=195)	28.15 (10.13)	106 (54.4)	8.76 (4.15)	56 (28.7)	37.31 (9.36)	84 (43.1)	45 (23.1)
St Andrews then Manchester (*n*=27)	27.96 (9.02)	16 (59.3)	10.22 (4.33)	12 (44.4)	42.37 (7.46)	18 (66.7)	9 (33.3)
Other than Manchester (*n*=6)	26 (7.77)	3 (50)	10.83 (4.92)	3 (50)	41.67 (5.32)	4 (66.7)	2 (33.3)
*p*	*ns*		*ns*		0.049		*ns*
Gender							
Male (*n*=124)	27.68 (11.01)	65 (52.4)	9.85 (4.86)	50 (40.3)	36.28 (10.16)	52 (31.3)	38 (30.6)
Female (*n*=232)	28.57 (9.56)	130 (56)	8.68 (3.68)	71 (30.6)	39.03 (8.83)	114 (68.7)	57 (24.6)
*p*	*ns*		0.014		0.008		*ns*
Year of study							
1 (*n*=141)	28.67 (10.36)	79 (56)	8.68 (4.30)	42 (29.8)	35.98 (10.14)	52 (31.3)	35 (24.8)
2 (*n*=77)	29.42 (9.72)	46 (59.7)	9.05 (4.11)	27 (35.1)	37.25 (9.78)	35 (21.1)	21 (27.3)
3 (*n*=66)	26.33 (9.69)	34 (51.5)	8.85 (4.08)	20 (30.3)	39.56 (7.84)	38 (22.9)	16 (24.2)
4 (*n*=47)	26.74 (10.08)	21 (44.7)	9.62 (4.18)	19 (40.4)	40.13 (7.60)	22 (13.3)	13 (27.7)
5 (*n*=25)	30.32 (10.29)	15 (60)	11.08 (5.28)	13 (52)	44.64 (6.02)	19 (11.4)	10 (40)
*p*	*ns*		*ns*		<0.001		*ns*

SD=standard deviation.

MBI cut-offs: high EE≥27; high DP≥10; low PA≤33; burned out if high in EE *and* DP; ns=not significance.

### Prevalence of burnout by age, gender, year of study, and institution

Chi-square analysis showed no significant impact of participant gender (*χ* ^2^ (1)=1.53, *p*=0.217), year of study (*χ* ^2^ (4)=2.75, *p*=0.600), or institution (*χ* ^2^ (3)=2.98, *p*=0.395) on the global prevalence of burnout in the sample. However, analysis of variance revealed that there were significant effects of participant gender, year of study, or institution on scores for the three components of burnout (EE, DP, and PA). The small number of participants who were in the St Andrews then Manchester cohort had significantly higher PA than those in the Manchester only cohort (*F* (3)=2.65, *p*=0.049). Males reported significantly higher levels of DP (*F* (1)=6.06, *p*=0.014) and significantly lower levels of PA than females (*F* (1)=7.04, *p*=0.008). PA scores increased with each year of study (*F* (4)=6.29, *p*≤0.001) (Table 2).

### Lifestyle and health behaviours by gender, year of study, and institution

#### Alcohol use

There were no gender differences in alcohol consumption. Mean alcohol intake scores were significantly higher in first year (46.86±13.92) than second year (40.46±9.98), (*F* (4)=4.48, *p*=0.002). Alcohol binge scores were also significantly higher in first year (38.47±11.41) when compared with second-year (32.44±8.68) and fourth-year scores (32.65±7.75), whilst third-year binge scores (37.45±11.97) were significantly higher than second-year scores (32.44±8.68), (*F* (4)=6.30, *p*<0.001). Alcohol consumption did not significantly differ between institutions.

#### Physical activity

Chi-square analysis indicated that there was a significant association between physical activity intensity (low, moderate, and high) and gender (*χ* ^2^ (2, *n*=325)=11.76, *p*=0.003), with a greater proportion of male students (*n*=59, 54.1% of males) in the high intensity physical activity group than female students (*n*=76, 35.2% of females). There was no significant relationship between physical activity intensity and year of study or institution. Physical activity levels differed by gender with males reporting higher weekly METs (2773.72±1708.57) than females (2214.12±1810.65), (*F* (1)=7.18, *p*=0.008). Reported weekly METs were significantly higher in fifth-year students (3500.46±2868.50) than in first (2138.96±1567.30) or second year (2331.78±1409.44), (*F* (4)=3.12, *p*=0.015). Physical activity levels did not significantly differ between institutions.

#### Diet

Gender impacted intake of several food groups. Female students reported lower FFQ savoury food consumption scores (13.85±9.23) than male students (18.33±11.31), (*F* (1)=16.22, *p*<0.001). Female students also had higher vegetable consumption scores (18.08±10.59) than male students (14.27±10.01), (*F* (1)=9.92, *p*=0.002). Vegetable consumption scores systematically increased with each successive year of study (*F* (4)=2.72, *p*=0.029) whilst sweet food consumption scores were significantly higher in third year (15.49±15.11) than in second year (10.01±8.03), (*F* (4)=2, 12, *p*=0.048). St Andrews only participants were found to have a significantly higher combined fruit and vegetable consumption scores (30.78±17.46) than Manchester only participants (24.83±14.96), (*F* (3)=4.78, *p*=0.003).

#### Smoking

Three hundred and sixteen (88.8%) participants reported that they did not smoke currently and had never smoked, 22 (6.2%) participants reported that they were ex-smokers, and 18 (5.1%) reported being current smokers. Chi-square analysis indicated that there was no significant difference between smoking status and gender, year of study, or institution.

### Relationship between burnout components, demographics, lifestyle, and health behaviours

Backward stepwise multiple regression analysis was used to test whether the demography, lifestyle and health behaviours of the students predicted levels of the three components of burnout (EE, DP, and PA).

The results of an 18-step regression using EE scores as the dependant variable (Table 3) indicate that four predictors explained 6.4% of the variance (*R* ^2^=.064, *F* (4,317)=5.36, *p*<.001). It was found that being in third year in comparison to first year (*β*=−0.13, *p*=0.020) significantly predicted lower EE scores. It was also found that having low levels of physical activity in comparison to high levels (*β*=0.15, *p*=0.005) and being an ex-smoker in comparison to having never smoked (*β*=0.14, *p*=0.012) significantly predicted higher EE scores. Sweet food consumption scores were important for the overall model but were not found to be a significant predictor of EE scores (*β*=0.09, *p*=0.081).

**Table 3:** Final model in an 18-step backward stepwise regression analysis predicting MBI emotional exhaustion (EE) scores

Variable (variable type/reference category)	*B* (SE)	95% CI	*β*	*p*
Constant	27.16 (0.87)	25.45; 28.87		
Year of study (categorical)				
Third year (reference: first year)	−3.36 (1.43)	−6.17; −0.54	−0.13*	0.020*
Level of physical activity (categorical)				
Low level (reference: high level)	5.62 (1.99)	1.69; 9.55	0.15	0.005**
FFQ Sweet Foods Score (continuous)	0.09 (0.05)	−0.01; 0.18	0.09	0.081
Smoking status (categorical)				
Ex-smoker (reference: never smoked)	5.81 (2.29)	1.29; 10.33	0.14	0.012*

*R* ^2^ for step 1=0.096, Δ*R* ^2^ for final step=−0.005 (final model *p*<0.001).

* *p*<0.05

** *p*<0.01.

A 17-step regression using DP scores as the dependant variable (Table 4) identified that four predictors explained 6.7% of the variance (*R* ^2^=0.067, *F* (5, 317)=4.51, *p*=0.001). It was found that attending St Andrews University only in comparison to attending Manchester University only (*β*=0.15, *p*=0.014), being male (*β*=0.12, *p*=0.041) and being in fifth year in comparison to first year (*β*=0.19, *p*=0.001) were significant predictors of higher DP scores. Being in fourth year (*β*=0.11, *p*=0.064) and savoury foods consumption (*β*=0.10, *p*=0.066) were important for the overall model but were not found to be significant predictors of DP.

**Table 4:** Final model in a 17-step backward stepwise regression analysis predicting MBI depersonalisation (DP) scores

Variable (variable type/reference category)	*B* (SE)	95% CI	*β*	*p*
Constant	7.17 (0.51)	6.17; 8.16		
Institution (categorical)				
St Andrews only (reference: Manchester only)	1.32 (0.53)	0.27; 2.36	0.15	0.014*
Gender (categorical)				
Male (reference: female)	1.05 (0.51)	0.04; 2.06	0.12	0.041*
Year of study (categorical)				
Fourth year (reference first year)	1.35 (0.73)	−0.08; 2.78	0.11	0.064
Fifth year of study (reference: first year)	3.06 (0.91)	1.27; 4.86	0.19	0.001**
FFQ Savoury Foods Scores (continuous)	0.04 (0.02)	−0.003; 0.09	0.10	0.066

*R* ^2^ for step 1=0.096, Δ*R* ^2^ for final step=−0.004 (final model *p*=0.001).

* *p*<0.05

** *p*<0.01.

A 15-step regression using PA scores as the dependant variable (Table 5) identified that four predictors explained 14.5% of the variance (*R* ^2^=0.145, *F* (7,317)=7.52, *p*<0.001). Being male (*β*=−0.14, *p*=0.009) was significantly predictive of lower PA scores. Being in third (*β*=0.13, *p*=0.020), fourth (*β*=0.14, *p=*0.010) or fifth year (*β*=0.24, *p*<0.001) in comparison to first year was significantly predictive of higher PA scores and the significance of the prediction increased with each successive year. Higher alcohol binge scores were significantly associated with higher PA scores (*β*=0.15, *p*=0.005). Having moderate (*β*=−0.12, *p*=0.027) or low levels (*β*=−0.17, *p*=0.002) of physical activity in comparison to high levels were significant predictors of lower PA score.

**Table 5:** Final model in a 15-step backward stepwise regression analysis predicting MBI personal accomplishment (PA) scores

Variable (variable type/reference category)	*B* (SE)	95% CI	*β*	*p*
Constant	33.99 (2.05)	29.95; 38.04		
Gender (categorical)				
Male (reference: female)	−2.92 (1.11)	−5.09; −0.74	−0.14	0.009**
Year of study (categorical)				
Third year (reference: first year)	3.13 (1.34)	0.50; 5.76	0.13	0.020*
Fourth year (reference first year)	3.99 (1.54)	0.96; 7.03	0.14	0.010*
Fifth year (reference: first year)	8.69 (1.95)	4.85; 12.54	0.24	<0.001***
AUQ binge score (continuous)	0.14 (0.05)	0.04; 0.23	0.15	0.005**
Level of physical activity (categorical)				
Moderate level (reference: high level)	−2.37 (1.07)	−4.48; −0.27	−0.12	0.027*
Low level (reference: high level)	−5.97 (1.91)	−9.73; −2.19	−0.17	0.002**

*R* ^2^ for step 1=0.162, Δ*R* ^2^ for final step=−0.005 (final model *p*<0.001).

* *p*<0.05

** *p*<0.01

*** *p*<0.001.

## Discussion

This study investigated the prevalence of burnout in UK medical students and explored the association between burnout and health related behaviours. Our findings indicate that burnout was present in this undergraduate medical student sample, with one in four participants being categorised as burned-out according to the appropriate cut-offs in the MBI (30). In addition, this study has indicated that specific demographic, lifestyle, and behavioural factors, including physical activity, sweet or savoury food consumption, alcohol bingeing, gender, year, and institution of study may predict medical student's experience of burnout components.

The results of this study show that burnout exists in this undergraduate medical student sample (26.7%), with a substantial number of participants reporting high levels of EE (54.8%) and DP (34%), and low levels of PA (46.6%). These results are consistent with a recent systematic review on burnout in medical students (5), and suggest that many medical students are at risk of, or are already suffering from, burnout well before they qualify as medical doctors. In comparison to dental students, where 10–22% report high EE (45, 46), 17% low PA and 28% high DP (45), the percentage of medical students in this sample experiencing high EE or DP and low PA are concerning.

In addition, a number of demographic factors were shown to predict participant's experience of burnout, including gender, institution, and year of study. Institution was found to have an impact on both DP and PA. Although institution did not significantly impact on DP scores when examined via ANOVA, regression analysis suggested that participants who attended St Andrews University (St Andrews only cohort: Years 1–3) were significantly more likely to have higher DP than Manchester University participants (Manchester only cohort: Years 1–5). Additionally, analysis of variance found that mean PA was significantly higher for participants who had progressed from St Andrews to Manchester (Years 3–5) than for those in the Manchester only cohort (Years 1–5). Associations have previously been found between differing learning environments and burnout in American medical students (10). There are differences in the learning approach adopted by the two institutions in this study which may influence the student experience, and therefore potential for development of burnout. However, it is also possible that students who select to apply for either course may vary prior to entry, and thus we cannot determine from our data whether this result is influenced by differences in student cohort per se.

Male participants reported higher levels of DP and lower levels of PA than females suggesting that, in this sample, males may be at greater risk of burnout than females. These findings are partially supported by a recent meta-analysis of multiple professions conducted by Purvanova and Muros (11) who rebutted the belief that burnout is more commonly experienced by female employees and that males appear more susceptible to DP. Work examining the gender differences in burnout prevalence within medical student samples is limited and inconclusive (5). Further study is therefore required to clarify the relationship between gender and burnout in the medical student population.

This study revealed that DP and PA were positively associated with year of study. These findings are supported by previous research. Dyrbye et al. found that DP and PA increased with year of study in American medical schools (35). This increase in DP as medical students progress through their training has potential implications for communication with patients (47) and patient outcomes (48). There was a negative trend between EE and year of study in the current study with EE decreasing from first to third year, although scores still remained relatively high. These findings are contrary to Guthrie et al. who identified a modest increase in EE scores with year of study in a sample of medical students from the University of Manchester, UK (49). Perhaps the most striking finding, however, was that over 50% of first-year students reported high levels of EE and over 40% reported low levels of PA. This suggests that a very large proportion of students are at high risk of developing burnout very early in their medical training and that there may be scope for a preventative intervention before burnout becomes established.

Previous studies in medical student samples have noted a change in the components of burnout with year of study and others have identified gender differences, however this study furthers our understanding of burnout by identifying a potential relationship between medical students’ lifestyle and health behaviours and the components of burnout. Interestingly, physical activity predicted lower EE and higher PA, while alcohol binge scores were predictive of higher PA. This previously unreported finding related to alcohol suggests that the relationship between alcohol consumption and psychological distress in not unidirectional (19, 21). There is some evidence that alcohol may be used as a coping strategy for stress in certain individuals (50, 51), however this is unlikely to result in the perceptions of high PA in this sample. Szmigin et al. (52) conducted interviews with young adults in the United Kingdom and proposed that young drinkers have a ‘hedonistic’ approach to binge drinking, suggesting that medical students may be drinking for pleasure rather than as a coping mechanism of stress and burnout.

Physical activity was the most predictive of all the lifestyle and health behaviour variables and was associated with higher PA and lower EE. This finding agrees with recent research suggesting that physical activity may be associated with a reduction in the experience of burnout in male workers (27) and in trainee doctors (28). Physical activity has been shown to be beneficial for mental health 53–57). Salmon (56) postulated that many aspects of physical activity, including an improved sense of self-control and greater social interaction, may have positive implications for mental health. The results of this study suggest that the active promotion of physical activity within medical student populations may help to protect students from the effects burnout.

Although not statistically significant predictors, sweet and savoury food scores were retained in the final regression models for EE and DP respectively. This suggests that there may be a relationship between diet and the components of burnout. Torres and Nowson (58) reviewed the literature exploring eating behaviour and stress and concluded that chronic stress may increase the consumption of such high-energy foods. Devine et al. (59) examined working conditions and dietary choices in working parents and found that long and busy working hours were associated with poorer dietary choices and missed meals. It is possible that participants in the current study who were experiencing higher levels of EE and DP were making poorer dietary choices as a maladaptation to their busy schedules and as a coping mechanism for their increased feelings of mental distress. As this study only collected self-report data the actual intake of the food groups cannot be confirmed, further study may clarify the specific relationship between burnout and diet choice.

In this sample, being an ex-smoker was significantly predictive of higher EE scores. There is little research examining the relationship between burnout and smoking; however, links have been suggested between occupational stress and smoking (60). A recent study has also highlighted that stress increases cigarette cravings (61). These results suggest that there is potentially a link between smoking behaviour and burnout. However, the number of smokers and ex-smokers in this study was low in comparison to non-smokers and therefore it is difficult to meaningfully interpret this finding; further study is required to fully understand this link.

There are limitations to this study. The response rate in this study was relatively low; this may in part be due to the timing of the data collection which was conducted during the pre-exam period. This low response rate must be taken into account when considering these findings, although it is possible that those students who were experiencing high burnout may have been less likely to complete the survey. In addition, the response rate was unequal between years and female students were over-represented in the sample. The population of medical students at the Universities of St Andrews and Manchester at the time of this study was 57% female. This slight over-representation of female medical students in our sample may have impacted upon the results and the unequal response rate between years of study reduced the scope for cross-year analysis. The number of students was greatest in first year where the prevalence of burnout was comparably lower than other years and this may have underestimated levels of burnout in this medical student sample. Although there were differences between the institutions in the burnout reported by participants, differences in course structure and content may influence the characteristics of the students who apply to and study at a particular institution, therefore caution must be taken when attempting to generalise these results to all medical schools.

## Conclusion

This study highlights the risk of burnout within all years of this medical student sample as well as the importance of understanding the various lifestyle and health behaviour factors that predict burnout in medical students. Although the amount of variance in burnout predicted by these health behaviours is small, this study suggests that they do play a role and that medical students should consider making healthier lifestyle choices early in their medical training, within their first year of study, to help prevent burnout becoming established. Particular attention should be paid to increasing physical activity, improving dietary choices and encouraging male medical students to engage in healthy behaviours. Future research should incorporate health behaviour choices within models of burnout in medical students to aid with the development of preventive interventions.
